# Multi-Agent-Based Urban Vegetation Design

**DOI:** 10.3390/ijerph17093075

**Published:** 2020-04-28

**Authors:** Ahmed Khairadeen Ali, Hayub Song, One Jae Lee, Eun Seok Kim, Haneen Hashim Mohammed Ali

**Affiliations:** 1 School of Architecture and Building Science, Chung Ang University, Seoul 06974, Korea; ahmedshingaly@gmail.com (A.K.A.); esk1129@cau.ac.kr (E.S.K.); 2 Haenglim Architecture and Engineering Company, 201, Songpa-daero, Songpa-gu, Seoul 05854, Korea; o.lee@haenglim.com; 3 College of Agricultural Engineering, University of Duhok, Duhok 42001, Kurdistan, Iraq; engineerhaneenhashim@gmail.com

**Keywords:** multi-agent system, visual algorithm, urban vegetation design, automatic modeling, computational design

## Abstract

Urban vegetation is an essential element of the urban city pedestrian walkway. Despite city forest regulations and urban planning best practices, vegetation planning lacks clear comprehension and compatibility with other urban elements surrounding it. Urban planners and academic researchers currently devote vital attention to include most of the urban elements and their impact on the occupants and the environment in the planning stage of urban development. With the advancement in computational design, they have developed various algorithms to generate design alternatives and measure their impact on the environment that meets occupants’ needs and perceptions of their city. In particular, multi-agent-based simulations show great promise in developing rule compliance with urban vegetation design tools. This paper proposed an automatic urban vegetation city rule compliance approach for pedestrian pathway vegetation, leveraging multi-agent system and algorithmic modeling tools. This approach comprises three modules: rule compliance (T-Rule), street vegetation design tool (T-Design), and multi-agent alternative generation (T-Agent). Notably, the scope of the paper is limited to trees, shrubbery, and seating area configurations in the urban pathway context. To validate the developed design tool, a case study was tested, and the vegetation design tool generated the expected results successfully. A questionnaire was conducted to give feedback on the use of the developed tool for enhancing positive experience of the developed tool. It is anticipated that the proposed tool has the potential to aid urban planners in decision-making and develop more practical vegetation planting plans compared with the conventional Two-Dimensional (2D) plans, and give the city occupants the chance to take part in shaping their city by merely selecting from predefined parameters in a user interface to generate their neighborhood pathway vegetation plans. Moreover, this approach can be extended to be embedded in an interactive map where city occupants can shape their neighborhood greenery and give feedback to urban planners for decision-making.

## 1. Introduction

As cities improve and expectations for everyday comforts increase, urban residents solicit higher standards for personal satisfaction and lodging conditions, creating more appeal to arrange and structure cities [1,2,3,4]. Urban tree planting offers a viable way to improve the weather comfort conditions in the climate-proof cities, especially in high-density compact neighborhoods [5], and reduce carbon emissions and its sequestration [6,7,8]. However, tree planting on the street faces difficult challenges to grow compared with trees planted in the parks [9]. Street trees are easily subject to stresses due to their proximity to atmospheric pollutants, poor drainage, inhospitable soil, mechanical damage, high and low ambient temperatures, and lack of space for growth [10,11,12]. These factors should also be considered in the selection of street tree species. Due to inadequate planting space and high costs of individual protection of trees, collision and vandalism have destroyed many street trees [13,14,15]. Tree planting plans require wide assent from urban planners. Therefore, understanding people’s perspectives is key to effective smooth usage of urban planning decisions [9,16]. Currently, vegetation in the urban fabric is a popular program, with the extreme target of improving the natural quality in urban territories, including pathways and road conditions [1,2].

Trees can bring different advantages, including the capacity to constrict noise levels [3,4], purifying polluted air of densely urban areas [13,15], and lessening urban stormwater overflow [17,18]. They can increase people’s prosperity by helping individuals decrease stress levels as they connect to nature [9]. Moreover, they can help mitigate the feeling of severity in jam-packed urban regions by bringing different advantages such as increasing the economy of the land surrounding the trees and providing a satisfying aesthetic impact [13,19]. Trees can also act as a barrier between pedestrians and moving vehicles, giving a nice enclosure feeling and increasing walkability [20]. In general, urban residents have a positive perspective on increasing vegetation in an urban environment [21,22,23].

Trees also have negative impacts when planted in an urban environment. For example, they might trigger hypersensitivities to pollen and dust [24,25,26] or become a habitat for insects [21]. Moreover, fallen leaves cause inconvenience and require constant cleaning. As opposed to the traditional recognition that tree planting can improve air quality, recent field explorations have found that planting in high-contamination territories (for example, “hotspots” such as traffic intersections and inside road ravines) may increase pollution at the ground level [8,24,27,28,29]. Therefore, there is a principal question of whether individuals would favor tree planting in urban areas in “all cases”, on the off chance that they think trees might cause further deterioration of air quality.

Various road and building designs have been studied; city residents prefer space between buildings and streets, and they loathe encased settings and blocked perspectives [9,30]. The size of street space has an extraordinary effect on daylight and ventilation; it is the connection between the proportion of street width and surrounding building height [31]. The vegetation scale and location in complex and high-density areas have not been designed well and cause many problems for city residents; therefore, a user-friendly design tool is needed to aid urban planning of street vegetation, which can provide three dimensional modeling, optimization of urban solutions, and technical drawings that can be utilized by urban planners and citizens without any programming backgrounds [9,22,32,33]. With a simplified design tool developed using visual programming language, residents can have a chance to participate in urban decision-making to leverage the city context and increase life quality [34,35,36,37,38].

This paper proposes a bottom-up approach for automatic modeling of pathway vegetation in urban contexts, in compliance with city planning regulations, environmental requirements, and best practices, by utilizing visual programming and generative design technologies. The bottom-up approach in this research refers to the piecing together of different modules to produce a vegetation design tool. To identify the advantages and limitations of the vegetation design tool, a system prototype was developed and verified. The main research question is how visual programming and automatic modeling can leverage street vegetation design more efficiently? Also, how can the environmental analysis simulations improve vegetation selection and design decision-making? The more specific objective of the study was to develop a user-friendly urban vegetation design, which can generate 3D visual models of plants in the urban context, technical drawings needed for planting on pathways, and quantity take-offs of these technical drawings. With the aid of a computation multi-agent system, this research also tested decision-making using an evolutionary generative design engine. Finally, a questionnaire was conducted to measure the practicality of the developed tool and give feedback on how to improve its user interface to be more user-friendly. This research work does not consider the entire greenery regulations enforced by the city forest planning department; it only considers articles that can be converted into mathematical equations at this stage.

## 2. Literature Review

### 2.1. Vegetation Practices in Urban Street Planning

Many researchers have studied vegetation in the urban fabric from different aspects such as selection, design, planting methods, and its impact on human behaviors. Only a limited number of species are planted as urban trees due to the lack of systematic trials of species and poor access to planting material, even though trials have shown some urban trees to be most hardy, aesthetically pleasing, and easy to propagate [39,40]. Often wild, edible plants with low-care requirements are excellent candidates for ornamental street tree planting.

Miller (1997) developed a model for selecting specific tree species that best fit an urban environment. Selection measuring criteria this researcher suggested include urban site conditions, aesthetic factors, and social factors [41]. Nevertheless, this researcher downgraded other factors, including tree growth scale, urban planning regulations, longevity, ease of cultivation, and mass propagation. These criteria were highlighted as important factors in the selection of tree species [32]. According to S. Pauleit [32], trees in the cities of the United Kingdom are very much undervalued, and only an afterthought in the process of planning, design, and management of streets, and their town’s level of expenditure for trees, street tree quality, and site preparation was developed recently [39,42,43].

According to the British standards [5], tree selection criteria include the location (choosing the site and assessment of constraints), tree selection (decision support and thinking long-term), ecosystem services (the benefits of trees), biodiversity (tree selection to enhance and support biodiversity), procurement (contract growing, and procurement policies and standards), planting and establishment (ground preparation, production system choice, mulching, weeding, watering, formative pruning), pests and diseases (threats, signs, symptoms, and management solutions), tree retention and removal (transparent decision-making, effective and timely consultation).

In many cities in regions such as those in Thailand and the Philippines, local business people have invested much in potted woody plants, which are planted in front of their shops or on roof gardens [44]. For instance, street trees are especially desired in business districts because local shop owners can advertise on tree protectors. Entrepreneurs generally prefer to sponsor trees in high-traffic central locations [33,45,46]. Street trees survive and flourish best when people living adjacent to them commit themselves to be responsible for tree care in one or other form. It can be anticipated, once the full or partial responsibility of trees by residents is institutionalized, the survival and viability of street trees will be increased dramatically. The relationship between tree size, building height, and urban street width still needs to be studied more in a 3D model environment, checking how they affect each other in visual and environmental aspects. Due to the reasons mentioned above, there is a need to integrate the tree planting in the early stages of urban planning to avoid conflict between traffic and pedestrian spaces and maximize their benefits by integrating the landscape architects with the team of urban planning [47].

### 2.2. Need for an Automated Urban Street Planting Approach

Researchers have developed artificial environment tools in which policy can be developed and tested to cope with the increased complexity of the current urban context [22,48,49]. During the last decade, Geographic Information System (GIS) has been created from generally clear frameworks for capacity, recovery, and introduction of spatial data towards frameworks that back complex spatial examination. The increment in computational control and the presentation of Cellular Automata (CA) expanded the expository capabilities of GIS for displaying complex energetic spatial forms [17,30,50,51]. The Urban Climate Map (UC-Map) is a handy tool for urban planning purposes since it integrates various urban climatic parameters with urban planning considerations. While this method has been used in Germany since the early 1980s, its wider adoption by other cities has been slow because planning and urban climate research arises out of different knowledge domains [52,53,54].

Computational programs have been developed to aid urban planners in making environmentally responsive design decisions in the early stages of the design process [52]. Visual programming has been utilized by urban planners to optimize and analyze urban contexts, such as Grasshopper in Rhinoceros that requires no background in programming and can rapidly conduct complex computational calculations [47]. Ladybug and Honeybee are two environmental analysis add-ons in the Grasshopper environment which provide analysis including daylight study, massing orientation study, radiation visualization, and analysis and energy modeling of a 3D geometry in different stages of the design process [47,48].

Using Screening Instrument for Domain Environment Assessment (STEVE) tool, urban planners’ can easily calculate the anticipated air temperature based on their plans and do a few plan changes when they experience weather changes [15]. Tree design and monitoring tools have already been developed to quantify and value ecosystem services provided by the tree in the urban context [42,55]. Vegetation detection and feature extraction using lidar and multispectral remotely sensed data have also been developed to check the ratio, species, and quantification of vegetation in the urban context [43,56]. These image processing and 3D scanning services are essential for vegetation monitoring purposes. However, the previously mentioned tools require prior training on the software and knowledge in urban planning for vegetation.

Subsequently, an elective instrument is found in Multi-Agent Systems (MAS) that offers a conceptual and methodological approach to incorporate agents into energetic spatial models of decision-making [57,58,59,60]. Itami and Gimblett (2000) simulated complex interactions between human movement and the outdoor recreation environment with Recreation Behavior Simulator (RBSIM2), coordinated with a GIS to be utilized as an assessment device for open space management [61,62]. Benenson (1998) simulated the flow in home designs in a large city context utilizing MAS [63]. Therefore, MAS offers alternative solutions in a short time with the power of computational design that helps urban planners in decision-making and choosing the best-fitting generated model for each specific condition. A new paradigm of collaboration between all city participants in the early stages of urban planning is arising. Both city occupants and planners become providers and recipients of the decision-making. The rapid development of new ways of use of the network has been observed in the last few years [49].

City rules, standards, and regulations need to be quantified and converted from textual format into a mathematical format to be integrated with automatic modeling algorithms or multi-agent systems. There have been advancements in converting text into a variety of output formulas, like the mathematical markup language [64] and mathspeak [65]. Therefore, an urban vegetation design tool is needed that includes city regulations, tree specifications, urban design planning rules, environmental analysis, and construction drawings all together in one platform.

## 3. Research Design and Framework

To understand the nature of greenery in the urban context, this study commenced with a detailed exploration of vegetation design and impact on the urban city fabric and previous literature. After that, the present status of urban vegetation planning, city forest policies, and state-of-the-art computational design and multi-agent systems was reviewed. Initial investigation of visual algorithms’ impact on urban planning motivated the development of a conceptual framework for automated urban street vegetation modeling. Hence, the proposed model was developed and implemented in a case study to test the system’s convenience and effectiveness.

A bottom-up approach was adopted in the prototype of trees design tool, where all surrounding constraints are considered from the beginning. This approach can help decision-makers by providing urban planners with solutions that are already optimized. Also, It can give feedback on how the resulting tree choice and position will behave inside the urban context when the tree grows [66]. The developed bottom-up strategy incorporates city rules and vegetation design regulations associated with (a) urban fixtures such as existing streets, buildings, benches, and pathways; (b) environmental analysis, such as Radiation Factor Analysis (DLA), Continues Daylight Autonomy (CDA), and field of view; and (c) construction simulation feedback that conditions the design process.

The proposed system evaluates the outcome design using two analytical factors. The first factor is the output model compliance with the rules derived from the city policies considered in this paper, tree specifications, and geographic constraints. The second factor is the environmental analysis that involves DLA for the amount of light passing through vegetation, especially trees, and CDA that measures the percentage of annual daytime hours occupied by the urban pathway per year [67].

**Figure 1:**
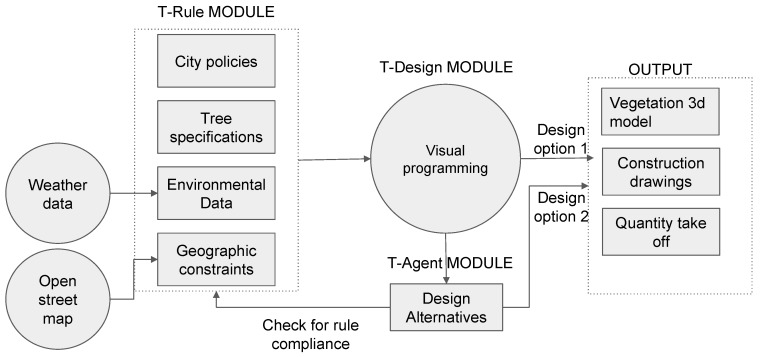
The conceptual framework for street vegetation design modeling tool.

The proposed approach, as illustrated in Figure 1, reflects the conceptual framework that proactively deals with street vegetation design tools. The conceptual framework for the multi-agent-based street vegetation design approach comprises three modules.

The first module is a rule extraction module called T-Rule. It consists of the extraction of vegetation-related rules and regulations from city policies and urban regulations, environmental weather analysis data, essential specifications for tree survival and growth, and geographic spatial data extracted from open street maps. The city policies used in this study are limited to the street vegetation rules of Seoul, South Korea, which were converted into mathematical equations to be used for output model evaluation purposes. The tree specifications consist of scale, growth, depth-to-height proportion, canopy size, and root-growth data of the trees that are used in this research. The environmental data are used for analytical purposes in T-Design (see below) and as Fitness Objectives (FO) in T-Agent (see below). The geographic constraints deal with the urban context surrounding the target pathway, such as buildings, vehicles, streets, and alleys.

The second module, T-Design, consists of street vegetation design tools in visual programming that convert urban planning rules in T-Rule to graphic algorithms in a single-script environment. This module automizes the design process in a hidden layer, with reduced user input, and makes it user-friendly to simply choose from a dropdown list or slide options given the design parameters. In this research, parameter refers to the design variable included in the User Control Panel.

The third module, T-Agent, is a multi-agent process that proposes alternative vegetation design scenarios depending on fitness objective criteria defined in advance. This module lets the program choose the best solutions for street vegetation design according to the same parameters given in T-Design to challenge the user’s decision and give insight to more urban vegetation design alternatives.

The outputs of the developed design tool are 3D rendered models of the pathway equipped with vegetation, 2D drawings for the technical construction of vegetation planting on the pathway, and quantity take-off of the vegetation trees needed for the construction drawings. T-Agent uses the commercially developed modeling software Rhinoceros and the Grasshopper plug-in as a platform to run the agent simulations [21,22]. Python and the Grasshopper visual programming language is used to control the multi-agent process, as illustrated in Figure 1.

## 4. Proposed Prototype System Based on Framework

### 4.1. Rule Extraction (T-Rule) Module

The T-Rule module, as shown in Figure 1, is built to develop a controlling and evaluation checkpoint for the visual algorithm process. This module includes logics and equations extracted from city policies related to vegetation in the urban context. The city targeted in this research is Seoul, South Korea. Several policies from the city regulations were converted from textual format into equation format using mathematical markup language, as illustrated in Table 1. The module also adopts vegetation rules and equations of the Australian landscape management standards, simplified and developed by the Trees Impact Group that converted rules from Australian tree planting specifications standards into simple mathematical equations [68].

This research only focused on rules that can be translated into logic and contain urban planning properties, such as the first three equations adopted from Management of Streets in Seoul planning rules, as illustrated in “Equations (1)–(3)”, where TDT is the distance between trees and PD is the distance between the tree trunk and street/building edge [70]. Diameter at Breast Height (DBH) is a significant descriptor of tree size and urban mass. It tends to be utilized to calculate the volume of soil that the tree will require for its long-term health. As a tree develops the base of its stem extends (trunk flare), and breadth of the essential roots increases, especially near the base of the stem. Significantly, trees are planted far enough from structures and other urban fixtures to permit them to normally grow, without potential harm to their surroundings and roots.

**Figure 2:**
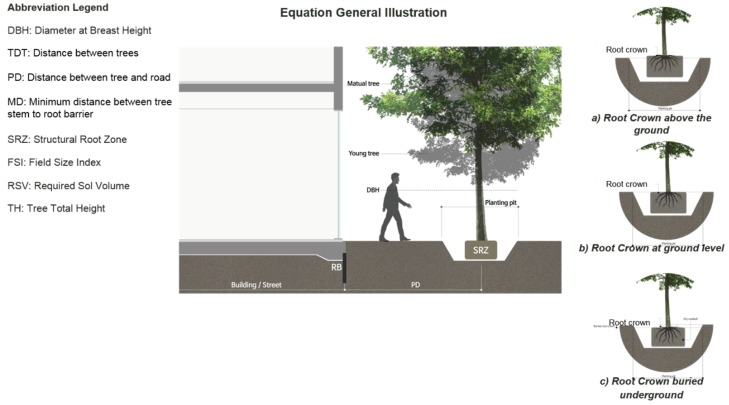
An illustration of T-Rule module rules and regulations including root crown positioning scenarios: (**a**) Root crown position above the ground level illustration; (**b**) Root crown level matches the ground level illustration; and (**c**) Root crown buried underground illustration.

Tree Impact Group converted tree specification rules to simple equations [68]. These rules focus on essential tree space needed to survive in an urban environment. For example, they calculated minimum tree pit depth and soil volum required for a tree to servive in urban environment regardless of its geographic location. These rules were adopted by this research and integrated into our modeling algorithm in Equation (4)–(10). Equations (4)–(6) calculate the planting distance to the nearest urban object, as mentioned in Table 1. Note that the PD generated in Equation (7) is for estimation only and is not precise. They are used only for guiding the street vegetation tool proposed in this paper. Equation (8) developed by the Tree Impact Group, covers the Australian Forest Standard (AS 4970-2009), where the Structural Root Zone (SRZ) is described as “the area required for tree stability.” This rule has been adopted by T-Rule as illustrated in Figure 2.

Trees planted in urban cities need to be sealed with Root Barriers (RB) to prevent adverse impacts on infrastructure. However, the size of a tree pit should be adequate according to tree type and size; otherwise, the tree might fall or wither. As observed in storm events, trees that fall pull their roots out with them if they are planted in a compromised box near urban infrastructure, which severely restricts their natural growth [71,72]. Therefore, T-Rule calculates the optimum SRZ for each tree species in each specific infrastructure location. Before considering installing RB, a minimum safe distance from a tree is calculated by this module. This distance is represented by a spherical safe space for the tree to survive, and the RB should be located outside this sphere. This logic can help designers and urban planners to allocate sensible tree spaces in the planning stage, as depicted in Equation (9) where the minimum distance (MD) from the stem center to RB equals to 3.5 multiplied by the DBH.

In urban environments, all spaces that objects occupy above and underground are measured and have a critical impact on each other; therefore, the amount of soil provided for vegetation planted on the streets should be calculated and considered in the street vegetation design tool. Conventionally, the urban soil is compacted and then distributed to tree pits, which often causes the soil chemistry to be less than required. A sufficient amount of soil should be provided for trees to survive in the long-term; otherwise, they might wither away or fail to grow due to insufficient urban soil.

To calculate the Required Soil Volume (RSV) a balanced formula based on the Australian Standard 2303:2015 has been developed by the Tree Impact Group as follows: A variation of Size Index, sometimes called the Field Size Index (FSI), equals to the total tree height in meters multiplied by the DBH measured in millimeters, as described in Equation (10).

The watering system for trees is out of the scope of this research. However, the irrigation of newly planted trees is critical for them to thrive. Most short-term watering problems are related to excessive or insufficient watering volume. Therefore, in the quantity taken-off of the trees after design, a data-sheet is generated that calculates the watering volume and frequency needed for each tree type covered in this research to guide the construction agent on how to irrigate or water the newly planted trees.

This research adopted tree classification made by Seoul vegetation management [70] that divided trees into three clusters depending on their height: Type A consists of tree families that are higher than 12 m; Type B consists of trees with heights ranging between 6 and 12 m; and Type C contains trees that are lower than 6 m, as shown in Table 2. This classification also considered the canopy radius and depth-to-height ratio of the tree for vegetation species selection. D/H of the tree (where D refers to the canopy diameter, and H refers to the height of the tree) is compared to the D/H of the street (where D refers to the width of the street and H refers to the height of the surrounding building). The D/H comparison between the tree and street is used as informative data that might guide the user to choose a suitable tree class from the three tree clusters mentioned in Table 2.

The right conditions for the “root crown” are essential for the long-term survival of a tree. The structure of the underlying roots of trees has developed to suit subterranean life. Thus, portions of the tree have advanced to live and develop over the ground. The intersection where those subterranean parts or ground parts meet is known as the “root crown”. It is preferred that the root crown is placed directly at ground level, as shown in Figure 2b. Be that as it may, it is normal for trees to be over-potted during the nursery stage—see Figure 2a—and/or planted too profoundly on location. Inappropriate root crown placement might harm trees and surrounding spaces, and cause problems including girdled roots, a mat of fibrous roots, sealing-off the rootball with site soils, and collar root. In nature, root crowns can be buried for a variety of reasons: trees might be planted too deeply; subsidence; trees might be planted too low on a sloping site, or root balls might be covered in silt as a result of watering, as illustrated in Figure 2c.

The T-Rule can only be applied on sidewalks inside Seoul, South Korea, because Seoul vegetation standards only have been included in this study. The Tree planting specifications from the Australia Tree Impact group have been adopted in this study to guide only the planting process as they have developed easy-use equations for quantifying SRZ, PD, and soil volume, as illustrated in Table 1.

Urban tree rules, including city rules, tree specifications, and urban context constraints, are converted from text into mathematical equations, then imported into the T-Design and T-Agent as informative and guide constraints to label produced models as “comply with the rule’s models” and “out-of-context models”.

### 4.2. Street Vegetation Design (T-Design) Module

**Figure 3:**
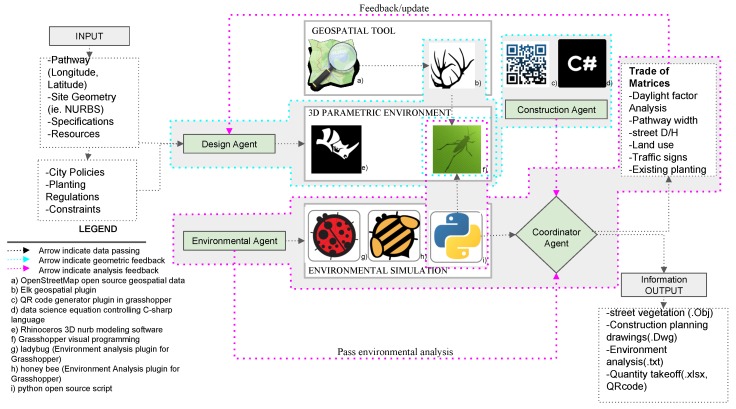
System Architecture for T-Design module.

T-Design has four main components: (a) design agent; (b) environment agent; (c) construction agent; and (d) coordinator agent, as illustrated in Figure 3. The design agent contains a set of design parameters initially defined by the user called User Control Panel as following: (a-1) target pathway, (a-2) Vegetation selection, (a-3) seating preferences, and (a-4) quantity take-off generation. The target pathway component of the design parameters obtains pathway, neighborhood, and street geometry from open street map managed by Grasshopper library called ELK—see Figure 3 (component b)—to control the geospatial data. The second subcomponent of the design parameters is the vegetation selection that contains a cluster of trees—see Table 2, shrubbery design preferences, and vegetation box dimension sliders. The third subcomponent of the design parameter is the seating preferences, where the user can customize seating bench design and dimensions. The fourth subcomponent of the design parameter is the quantity take-off tab where the user can generate construction data, design specification tables, and Quick Response (QR) code generator. This agent generates alternative street vegetation design scenarios based on user input and constrained by the urban context, city regulations, and vegetation planting equations imposed by the construction agent.

The environmental agent is used as an informative analysis tool to give insight on environment comfort within the pathway before and after each suggested vegetation design. The construction agent’s objective is to find the optimal urban design solution for street vegetation within the given multiple objectives. The environmental agent’s objective is to minimize the lux values of CDA while increasing the comfort daylight passing through the vegetation, especially trees, and measuring its effect on the surroundings. The coordinator agent controls the communication between the agents, process completion, and data passing.

The construction agent contains a set of rules extracted from the T-Rule module and uses these logics to check whether the 3D model generated by the T-design system is compatible with the rules. It gives a feedback error to the system to avoid these cases. It also generates technical drawings of the vegetation planting pit space explained in the PD equation, dimensions, geographic locations, quantity, and soil needed for the pit, calculated according to Equations (8)–(10). Also, it generates a unique ID—see Table 2—for each tree. Furthermore, it also produces some recommendation guideline reports associated with each specific tree species for the construction site to aid the planting worker with maintenance, watering methods, and quantity.

In this research, the “radRose” component inside the Ladybug library (see Figure 3, component h) was used to calculate the field of view of pedestrians while walking on the pathway with different tree distribution scenarios [19]. This view analysis component takes a user-determined visual plane, a field of view edge, and a separation breaking point to test for view obstruction from input geometries in our case buildings and vegetation. The outcome is a percent of unhampered perspectives and a progression of view cones with related distances and angles, which show the extent of visibility from a given location. This analysis allows the user to figure out how much the pedestrian sees or how much the street vegetation is blocking the field of view. This analysis can also be used to calculate if the tree or vegetation could block traffic signs for vehicles when it grows, to prevent such blockage in the planning stage.

The terms of metrics were developed to measure the quantitative assessment of the produced model’s performance. Metrics include daylight gain factor analysis, pathway width, street Depth-to-Height D/H, land use, traffic signs, and existing planting. Terms of metrics are provided to support the user in the decision-making while choosing the parameters of the vegetation model. The predefined metric assessment measurement tool is categorized under the coordinator agent, as illustrated in Figure 3.

The T-Design module outputs are 3D models of street vegetation in .Obj format. The 3D models contain urban geo-fabric model buildings, street, pathway planted with a tree, shrubbery, vegetation box, and seating bench depending on the user’s design parameter selections.

To coordinate the construction phase of tree planting, each tree is assigned a unique Identification Data (ID) that corresponds with a QR code. The QR code is a Two-Dimensional (2D) barcode that represents the tree ID with black and white dots. Some other data for construction can be stored inside tree barcodes, such as the spatial coordinates, tree type, tree ID, and required soil volume. The QR code can be attached to the tree before logistic operations start and provides information to construction laborers on each tree’s unique information at any time simply by scanning the code and using Application Program Interface (API) of the QR code generator tool [22]. The module also generates environmental analyses of the selected model including radiation analysis heatmap, daylight gain per hour heatmap, and field of view analysis data, as depicted in Figure 3.

### 4.3. Multi-Agent Alternative Generation (T-Agent) Module

The T-agent Module was built as a multi-agent script to optimize the best solution for vegetation design, with the potential to replace human decision-making in the vegetation design process or tree selection by the implementation of a metaheuristic search algorithm developed in an evolutionary solver engine. The generative agent receives initial design inputs depending on geographic area, environmental analysis information, and construction compatibility rules, acting as a multi-objective design tool.

**Figure 4:**
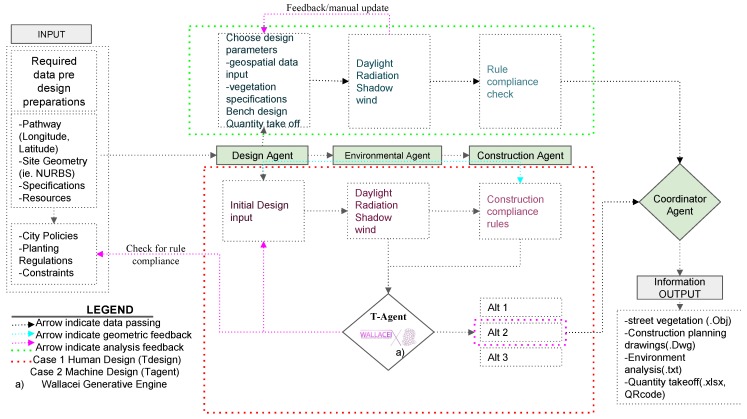
An illustration of the T-Agent design tool’s data process workflow and its comparison to T-Design decision-making process.

The initial design inputs are as follows: According to the neighborhood D/H, the system chooses a specific tree class. It assigns Type A class trees for high-rise building neighborhoods, Type B class for medium-rise building neighborhoods, and Type C class for low-rise building neighborhoods. As such, the system chooses a species from a specific class according to the building D/H. The rest of the design parameters are selected by T-Agent to best fit its objectives, as illustrated in Figure 4. The environmental agent information is inputted into the system as follows: minimize radiation gain on the pathway in summer; minimize daylight gain (increase shadow) on the pathway in summer; maximize radiation gain on the pathway in winter; maximize daylight gain on the pathway in winter [7,20,25].

The other objective is enhancing the field of view of pedestrians to increase walkability [11,57,73]. T-Agent discards any alternative that does not comply with the T-Rule module. This module uses the Wallacei evolutionary simulation engine, a plug-in in the Grasshopper environment. This tool can include multiple fitness objectives in its decision-making process [74]. It feeds the initial results back into the design agent and generates the best-fitting alternatives that comply with the T-Rule module rules and are closest to the objectives. T-Agent then runs the construction simulations and finds the optimum solutions given the various objectives. Based on the trade of matrices, the produced models are ranked according to their best compliance criteria with the simulation objectives and then passed onto the coordinator agent. The output 3D model alternatives are then saved as .obj format and associated with the output list as depicted in Figure 4.

## 5. Experimental Run

### 5.1. Case Study

**Figure 5:**
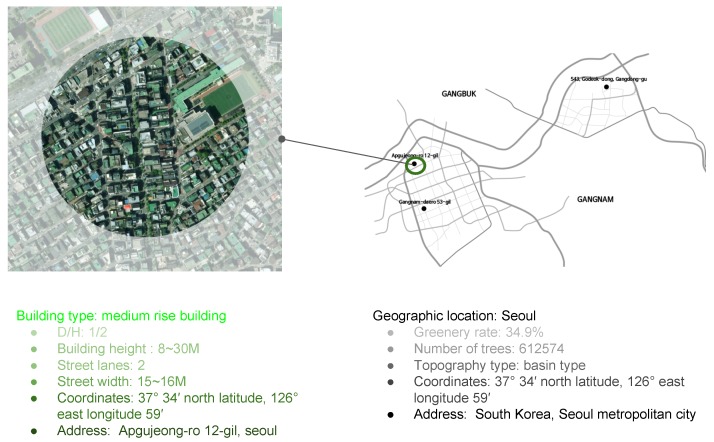
Case study’s geographic location and targeted zone area to apply the developed tool on its pedestrian pathway.

To test the T-Agent system methodology, a site with a medium-rise building type in Apgujeong-ro 12-gil, Seoul, South Korea was chosen as a case study. The site has the following specifications: D/H is ½; the building height is between 8 and 30 m; there are two street lanes, and street width is 15–16 m, as described in Figure 5. The pathway of this neighborhood was chosen to be run on the T-Design framework, a prototypical tool, in conjunction with the environmental performance to generate a street vegetation model.

**Figure 6:**
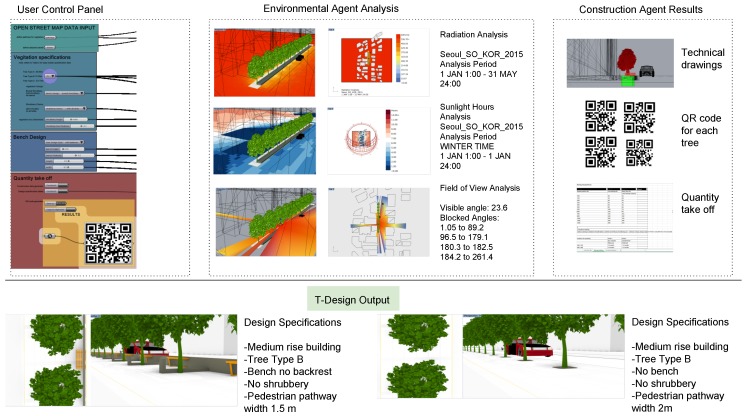
Output analysis and results of the T-Design tool applied to the case study showing the user control panel interface, environmental analysis, construction outputs and the visualized three-dimensional (3D) models of two different configurations.

The system workflow steps are as follows:

1. The user sets input parameters and initializes the design agent runs. The system implements the environmental agent to find the optimal tree positions. At each iteration, the system produces a unique model where the position of trees provides comfort daylight to the surrounding. The produced models are passed onto the DLA and CDA for lighting analysis.
2. When the environmental analysis is completed, the produced models are ranked and passed onto the construction agent to apply the regulation equations terms of metrics. Then, it produces the technical drawings for the construction planning stage.
3. The construction agent assigns equation logics to control different variables and defines the heuristic function as follows: (a) Equation (1) and Equation (7) control the distance between trees; (b) Equations (2) and (3) calculate the distance between the tree root crown and the street centerline depending on the tree type; (c) Equations (4)–(6) cluster the trees depending on the D/H of the neighborhood buildings and calculates the diameter at breast height; (d) Equation (8) calculate the SRZ needed for each tree type cluster in correspondence with the infrastructure or restriction defined by the user; (e) Equation (9) installs the RB at the minimum distance between the street and the tree stem; (f) Equation (10) calculates the soil volume needed for each tree cluster type defined by the previous equations as illustrated in Table 1.
4. The coordinator agent outputs are street vegetation design model, construction planning technical drawings, environmental analysis, and quantity take-off as shown in Figure 6.

**Figure 7:**
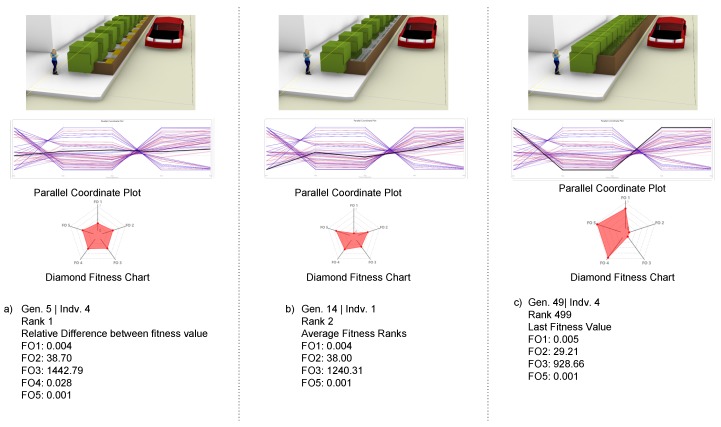
T-Agent revolutionary generative simulation agent alternative result 3D visualization models associated with its analytic parallel coordinate Plot and Diamond Fitness Chart: (**a**) Rank 1 produced model by T-Agent with it’s detailed Fitness Objective (FO) values illustration; (**b**) Rank 2 produced model by T-Agent with it’s detailed FO values illustration; (**c**) last rank produced model by T-Agent with its FO values illustration

The objective of the T-Agent is to initially search the generated solutions for design aesthetics and environmental efficiency based on different configurations and variable design parameters. The most efficient results are passed onto the construction agent to test model feasibility. Before starting the T-Agent calculation, the tree geometry, which contains many vertices, especially on the leaf, was replaced with bounding boxes containing a lot fewer vertices to reduce unnecessary computation time and get results faster without the system crashing. The feedback loop, at each iteration, modifies and optimizes the inputs into the system for heuristic results by running 50 cycles with 10 individuals per generation to produce 500 alternatives ranked according to the given fitness objectives. The cycle took about 7 h to finish with standard GPU computation power. As illustrated in Figure 7, the T-Agent generates a 3D model for each individual in the simulated generation associated with its parallel coordinate plot and diamond fitness chart for evaluation. An individual model, which is Generation 5 (individual 4) is ranked number one solution using the related difference between fitness values classifier offered in Wallacei multi-agent engine. As depicted in Figure 7 component (a), the parallel coordinate plot shows the balance between all five fitness objectives in this solution. The Rank 2 solution is fitting more to the winter environment than the summer environment, as it can be seen from its diamond fitness chart and FO values, as shown in Figure 7 component (b). The Rank 499 generated solution is the last ranked solution—see Figure 7 component (c)—as it is leaning toward succeeding in one fitness objective while neglecting another one, as it is shown in the parallel coordinate plot. The best-ranked solutions are then baked into the Rhinoceros 3D environment as (.obj) layer for further analysis.

The context as the list contains geometry that could block sunlight to the test geometry (in our case, pathway). The research scope is limited to pathway comfort characteristics using street vegetation. Therefore, to measure vegetation impact on a pathway, first, objects surrounding the pathway, including buildings, street signs, and electric pools, were imported into the algorithm as context geometry. Then, for radiation analysis, sunlight hours’ analysis in summer and winter were measured to compare the results with the pathway environmental analysis after vegetation, as illustrated in Table 3 (Context-1).

In the second analysis (Context-2), vegetation including shrubbery and trees, and seating benches were added to the surrounding context. Environmental analysis and field of view (visibility) were calculated to compare them with Context-1 and see the environmental and comfort impact of vegetation on the pathway (before and after vegetation).

The third analysis (Context-3) only considered detailed vegetation impact analysis on the pathway without considering the surrounding context. By isolating vegetation, a researcher can measure and check the individual environmental analysis for each design element, such as tree species or shrubbery size, on the pathway and simulate environmental analysis in different periods of the year to choose the best vegetation from the city index for each specific pathway.

The environmental analysis of the pathway was measured before planting (Context-1) and after planting (Context-2) to measure the environmental impact of the trees on the pathway, as shown in Table 3. This analysis can inform the user in choosing a proper tree species and distribute them more appropriately according to these analyses.

The field of view angle was measured in all three contexts to check which one has a significant impact on the human perspective. Perspective location is in the center area of the pathway with a human eye height of (1.6 m) on the pathway area. The visibility analysis uncovers the knowledge of the transparency value of the pathway while equipped with trees to aid the urban planner in how much the PD should be for the human visibility comfort rate.

**Figure 8:**
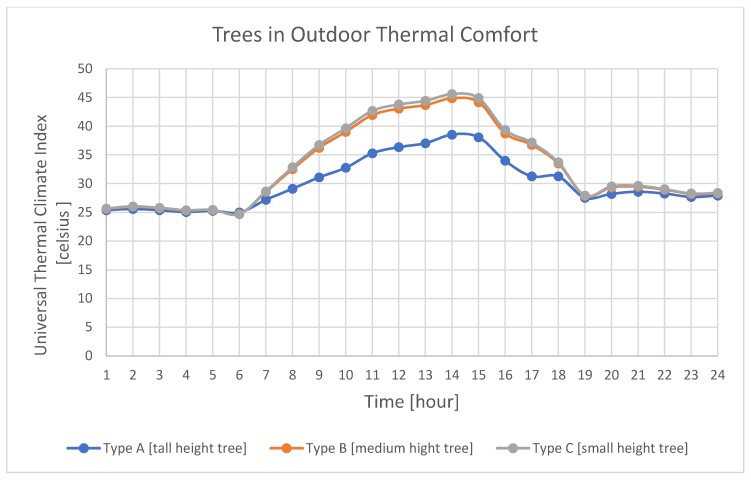
Trees in Outdoor Thermal Comfort measurements according to tree type classes.

To assist users to choose a suitable tree type—see Table 2—and species for the targeted urban context, a plot was added to the environmental agent to measure tree shading coefficients and tree transparency using Universal Thermal Analysis Index (UTCI). This plot analysis shows tree transparency in thermal comfort calculations for type A (tall, slender growing species), type B (medium-height trees general species), and type C (small-height trees), as mentioned in Table 2. As shown in the above plot, the type C tree class contains the closest thermal comfort values to 20 Celsius, defined by the UTCI, which makes it a good choice for this pathway, while type A and B have higher thermal comfort, as illustrated in Figure 8. The thermal comfort impact difference is small between type A and type B class of the trees because this research only included the pathway area in the calculation and does not measure the thermal effect of trees on the surrounding buildings. The user can choose either type A or B class and it will not have a big difference in the thermal comfort environment of the pathway. The detailed calculations of pavement material (dry sand, moist sand, mud, concrete, asphalt, and solid rock) and tree canopy transmittance affect the thermal comfort values shown in the above plot and can be changed in the T-Design—environmental agent to compare their impact on the tree species selection.

### 5.2. T-Design Tool Usage Feedback Questionnaire

The main purpose of this experiment is to test the usability of T-Design tools developed in this study. Therefore, the main research question for the experiment is “Is the developed tool user-friendly and can be used by citizens with no urban planning background to produce 3D models of urban vegetation?”

To address developed tool practicality, the study conducted a feedback questionnaire by letting participants use T-Design tools and choose vegetation design preferences from the user control panel—see Figure 6—and asking them to assess the usability and results of the tool.

In the beginning, the authors explain the tool objectives and software using instructions in group and individual, which took an average of 4 min. Then, the participant used T-Design and produced 3D mode and quantity take-off files that consumed an average of 5 min. Finally, the participants gave feedback by answering research questions, which took an average of 4 min.

Control variables included socio-demographic variables relevant to this study, like age, gender, education, and current city residence. In total, 20 individuals participated in this questionnaire. Most of the participants were male gender university undergraduate and graduate students in their 20s living in Seoul city, as shown in Table 4.

Table 5 Illustrates the mean and standard deviation of participants scores (5 point Likert scale) for seven questions. Question 1 measures if the user control panel is user-friendly and whether each design parameter contains enough description to guide the user on how to use it correctly. Most of the participants had positive feedback and scored an average of 4.6. Questions 2, 3, and 7 were designed to measure whether citizens with no background can use T-Design tool to produce vegetation 3D models and use this tool to give opinion to urban planners of their preferred urban vegetation configurations. Most of the participants had positive feedback and stated that the tool interface is simple and can be used by residents. Questions 4–6 were dedicated to measuring the technical process of generating quantity take-off, QR code, and viewing the environmental analysis. Most of the participants preferred the 3D model visualization of the urban environment surrounding the pathway.

Three more questions were added to the questionnaire to collect challenges participants faced, improvement suggestions, and additional comments. In the challenges part, most of the participants asked for more clear descriptions of each design parameter, therefore the authors modified the user control panel with more description and illustration to guide users throughout the T-Design workflow. Participants suggested adding another tab of tree species where users can choose specific trees and visualize it in the 3D model. They also recommended adding more control on having more than one tree type on one pathway to add variety to the tree designs. Detailed questionnaire data can be found in the supplementary materials section. 

## 6. Discussion

The case study revealed that the algorithm could produce design alternatives that adapt to the surrounding environment. The tool has successfully avoided any design iteration that is not compatible with the coordination agent embedded equations—see Table 1—and formed vegetation including shrubbery and trees alongside the pathway according to the user-preferred parameter selection. Before starting the T-Design, the process of choosing the targeted pathway consists of importing a closed curve or surface of the pathway manually to the algorithm and could be improved to simply pick a pathway form the map in the future. T-Design was able to produce 3D models of the trees, shrubbery, and install seating benches according to the predefined design parameters. It also generated technical drawings in a short period.

T-Agent was able to produce 500 alternatives compatible with the planning regulations and successfully ranked them from the best-fitting objective to the last. To decrease the multi-agent cycle run computation, the input phenotype of the tree element 3D model was imported as bounding box representation only because the leaves of the trees contain many vertices that make use of unnecessary computation. Tree’s full rendered geometry could be considered in T-Agent calculations instead of bounding boxes for better visualization to help the user read the produced model easily, but this will increase the time needed to produce the models. The T-Design was able to choose the correct classification of the trees—see Table 2—depending on the case study D/H. The multi-agent system produces the alternative solutions of 3D models and ranks them according to their best fitting to fitness objectives automatically. However, the process of extracting the best solution from the optimizer and printing it on the virtual environment canvas is still manual and can be automated in the future. The T-Rule included limited rules from city policies, urban context regulations, and tree planting specifications, and it can be expanded to include more regulations to guide the T-Design tool to produce more rule compliance vegetation design models. The T-Design tool could be extended to consider the land use of the urban context and measure how it will affect the vegetation design model outputs.

The developed tool is an open-ended algorithm that can be applied to any geographic location inside Seoul, and any step of the algorithm can be changed anytime, and it will affect all the other subsystems. However, in current conventional GIS software like ArcGIS, the tool is close-ended. In more details, produced model in this kind of software is static and user cannot go back to its history and alter the model parameters. Here comes the power of parametric algorithms versus close-ended conventional GIS software. Also, ArcGIS needs prior skill and knowledge in how to run the program, on the contrary, the developed tool in this research only needs the user to choose design parameters, and the tool then automatically generates visual models of rule-compliance vegetation in the urban context. Finally, to reproduce the models in T-Design and T-Agent, the complete algorithm data and instructions are included in the supplementary materials section.

## 7. Conclusions

Despite the vital development in urban planning, the tree planting locations and species selection are yet uncontrolled which has a significant impact on the pedestrian pathway. To address this issue, a multi-agent system urban vegetation design tool was developed and successfully tested with a pilot study that provided the intuition of how one design parameter in an urban context can affect the output generation and the behavior of the system. Based on the findings, the vital benefits are summarized as follows:

1. The research depicted that the developed tool has ample potential to enhance the urban vegetation planning by producing designs that comply with city regulations and tree specifications. In addition, citizens can generate their own pathway vegetation automatic 3D models without prior knowledge in urban planning, which helps to narrow the gap between the urban planners and occupants and helps the citizens engage more in vegetation awareness and shape their city.
2. Multi-Agent system generative 3D modeling of the pathway vegetation with its environmental analysis, construction drawings, and quantity take-off is another contribution of this study. It is expected that the T-Agent can generate many different types of alternatives to meet city regulations and assist decision-makers in developing more efficient urban vegetation plans that comply with the complex metropolitan city requirements.

The conducted questionnaire on the developed tool revealed that the user interface is simple and can be used by citizens. However, each design parameter needs more descriptions and illustration on how to use it. In addition, questionnaire respondents suggested adding a new design parameter where the user can choose tree species and including more control on the tree variations in a pathway rather than choosing only one tree type per pathway.

In the next step, a fully automatic system will be developed. The constructability ranking that is fed back into the system needs to be fully developed to produce a better version of street vegetation using heuristic functions. Each individual parameter needs to be separately examined by running iterative simulations for each, and its behavior and impact on the system should be checked. Finally, the system should be tested on existing street vegetation to compare its functionality with the current conventional street vegetation design tools.

Local citizens could test the proposed design tool, which will provide valuable insight into how the tool’s future direction might be. The citizens’ involvement in shaping their neighborhood’s vegetation and giving their opinion to the urban planners might help in the decision-making process and production of more fitting urban vegetation models. Since the developed study only extracted rules and standards from Seoul vegetation policies, it is only limited to these geographical locations and might not be compatible with other cities. Therefore, in order to extend the tool application to other geographic areas, customized rules of another city’s vegetation can be imported to the T-Rule module.

To enhance T-Agent tools, more data analysis and a case study is needed to refine further the heuristic function and the workflow between different agents. This approach can be extended to be embedded in an interactive map where city occupants can select a specific pathway and start choosing their planting preferences, then use it as feedback to the urban planners for decision-making.

## Figures and Tables

**Table 1:** Rules and standards related to urban street vegetation

Sources of Rule Legislation	Objective	Equation Number	RULE	Rule No.	Standard	Explanation
Rules from Gov	Distance between Trees (TDT)	(1)	TDT = 6~8 m	Act on the Creation and Management of Streets in Seoul Article 7	Criteria of Planting Materials of Garosu District, Management of Streets in Seoul, South Korea	The distance of the plants is based on the 6~8 m rule
	Distance between tree and road (PD)	(2)	Min. PD = 1 m	Chapter 2 (Creating a street tree) Article 4	Vegetation Reposition Location	Minimum distance from the road and pathway border to the center of a street tree ≧1 m
		(3)	PD ≥ 2 m			Plant a tree on the road without a sidewalk ≧2 m from the edge of the road
Rules from standards	Calculate Diameter at Breast Height (DBH)	(4)	Type A: DBH = 2.5% expected tree height ${}^{a}$	Australian Standard Appendix D: 2303:2015	Tree stock height and calliper	DBH estimates suggest that DBH, as a percentage of tree height, varies from 2.0% to 3.0% for tall, slender, growing species through to 5%–6% for stockier/thick-stemmed species, with general species somewhere in between
		(5)	Type B: DBH = 4% expected tree height ${}^{a}$			
		(6)	Type C: DBH = 5.5% expected tree height (TH) ${}^{a}$			
	Calculate PD according to DBH	(7)	PD=3.5×DBH.	Australian Standard for the Protection of Trees AS 4970-2009	Development Sites	PD is measured from the center of the planting pit
	Structural Root Zone (SRZ)	(8)	$SRZ\;radius={\left(PD\times 50\right)}^{0.42}\times 0.64$		the area required for tree stability	Root Zone in AS4970:2009
	Calculating Minimum Distance (MD) between tree steam and Root Barriers (RB)	(9)	MD=3.5×DBH	AS 4970:2009	Protection of trees on development sites	-
	Calculating Required Soil Volume (RSV) and Field Size Index (FSI) Needs of Trees in Urban Situations	(10)	RSV(m3)=FSI/100 FSI=TH(m)×DBH(mm)	Australian Standard 2303:2015	Balance formula found in the National Building Specification (NATSPEC) for landscape trees [69]	-

${}^{a}$ Use estimates for maximum height for trees in urban environments.

**Table 2:** Seoul tree classification depending on tree height.

Tree Class	Tree Height	Species Included	Remarks
	entry 1	data	data
Type A	>12 m	*Maidenhair tree*, *Metasequoia glyptostroboides*, *American sycamore*	Tall, slender growing species
Type B	6~12 m	*Yoshino Cherry, Castanopsis sieboldii, Japanese maple*	Medium-height trees general species (will apply in most cases)
Type C	<6 m	*Tetradium daniellii, Crepe-myrtle, Japanese camellia*	Small-height trees classified by the Municipality of Seoul

**Table 3:** Urban Context iteration effects on the targeted pathway environmental and viability analysis.

	**Analysis Period**	**Context-1**	**Context-2**	**Context-3**	**Vegetation Environmental Effect Rate Difference between Context-1 and Context-2**
pathway	-	Surrounding objects	Surrounding objects and vegetation	Vegetation only	-
Average radiation analysis result in $\left(Kwh/m^{2}\right)$	(summer) Jun-1- 12:00 to 13:00	Average: 1.73 Total = 334.15	Average = 1.06 Total = 204.48	Average = 1.12 Total = 216.76	−38.73
	(Winter season) Jan-1- 12:00 to 13:00	Average = 0.89 Total = 171.70	Average = 0.46 Total = 88.08	Average = 0.49 Total = 93.95	−48.31
Sunlight hours analysis (*hours*)	(summer) Jun-1- 01:00 to 24:00	Average = 6.11 Total = 1179.24	Average = 3.10 Total = 599.36	Average = 6.27 Total = 1210.74	−49.26
	(winter) Jan-1- 01:00 to 24:00	Average = 2.01 Total = 386.65	Average = 0.84 Total = 162.53	Average = 3.68 Total 711.25	−58.20
Field of view (visible angle in degrees	-	27.10	12.66	206.32	−53.28
Area occupation in the pathway for pedestrians in meter square	-	192.61	104.76	104.76	−45.59

**Table 4:** Descriptive statistics of control variables.

Control Variables		Percentage of Participants	Number of Participants
**Age**	21–30	80%	16
	31–40	20%	4
**Gender**	Female	10%	2
	Male	90%	18
**Education**	University	50%	10
	Grade School	50%	10
**Current city resident**	Seoul	90%	18
	others	10%	2

**Table 5:** Means and standard deviation of scores for dependent variables by the T-Design tool.

**Number**	**Question Statements**	**Mean** **(Standard Deviation)**
**1**	Overall, T-Design tool is easy to use	4.6 (0.50)
**2**	Citizens with no urban planning background can use this tool	4.2 (0.83)
**3**	I want to use this tool to give feedback to urban planners about my neighborhood vegetation design	4.35 (0.67)
**4**	Environment analysis helped me understand the effect of vegetation on pathway	4.55 (0.69)
**5**	I produced vegetation Quantity take-off and QR code easily	4.8 (0.41)
**6**	Visual quality of the 3D model was good	4.35 (0.88)
**7**	T-Design can improve citizen’s awareness of urban vegetation importance	4.35 (0.75)

## Supplementary Materials

Multi-Agent System Based street vegetation tool results along with future updates can be reproduced by the release script at https://github.com/ahmedshingaly/T-Design. In addition questionnaire detailed data conducted in Section 5.2 can be found in the google form link https://docs.google.com/forms/d/1bMG8viDO3aAiUG0_7KdiNcVf83lNf0QQeuFBcBZTzpo/viewanalytics.
